# Manifestations of HbSE sickle cell disease: a systematic review

**DOI:** 10.1186/s12967-021-02931-1

**Published:** 2021-06-16

**Authors:** Ibrahim Khamees, Fateen Ata, Hassan Choudry, Ashraf T. Soliman, Vincenzo De Sanctis, Mohamed A. Yassin

**Affiliations:** 1 Department of Internal Medicine, Hamad General Hospital, Hamad Medical Corporation, PO BOX 3050, Doha, Qatar; 2 Department of Internal Medicine, Faisalabad Medical University, Faisalabad, Pakistan; 3 Department of Paediatrics, University of Alexandria, Alexandria, Egypt; 4 Paediatric and Adolescent Outpatient Clinic, Quisisana Hospital, Ferrara, Italy; 5 Department of Medical Oncology/Hematology, National Centre for Cancer Care and Research, Hamad Medical Corporation, Doha, Qatar

**Keywords:** Sickle cell disease, SCD, Hemoglobin SE, HBSE, Sickle genotype

## Abstract

**Background:**

Sickle cell disease (SCD) is commonly encountered in Africa and Middle Eastern countries. The causative mutation in the gene encoding the hemoglobin subunit β (HBB) leads to various genotypic variants of the disease. This results in varied phenotypes, with a spectrum of complications, from benign to fatal. Hemoglobin SS (HBSS) genotype is associated with most of these complications; hence, it is a severe form of SCD. On the other hand, rare genotypes such as hemoglobin SE (HBSE) are considered benign. There is limited literature about the clinical manifestations and characteristics of patients with HBSE. We pooled all available data describing the phenotypic manifestations of HBSE heterozygote worldwide to perform a systematic review.

**Methods:**

We performed a systematic review according to PRISMA guidelines using PubMed, SCOPUS, and Google Scholar databases. Two independent reviewers (FA and IK) evaluated studies for eligibility and extracted data. We synthesized data on demographics, manifestations, and management of HBSE disease. PROSPERO Registration Number: CRD42021229877.

**Results:**

We found 68 HBSE patients reported in the literature. 24 cases were extracted from case reports whereas 44 cases from case series and retrospective studies. Turkey reported the highest number of patients (n = 22). 32 (47%) of the patients were males. The mean age was 20.9 ± 18.26 years. The mean HBS and HBE percentages were 61.1% ± 7.25% and 32.3% ± 5.06%, respectively, whereas the mean hemoglobin was 11.64 ± 1.73 g/dl. Reported manifestations of HBSE disease included acute vaso-occlusive pain crisis (n = 22, 32.3%), splenomegaly (n = 11, 16.1%), hemolytic anemia (n = 10, 14.7%), infections (n = 8. 11.7%), bone infarction (n = 4, 5.8%), gallstones (n = 3, 4.4%), venous thromboembolism (VTE) (n = 2, 2.9%) and stroke (n = 2, 2.9%), and hematuria (n = 2, 2.9%). Death due to HBSE complications was reported in three patients.

**Conclusion:**

HBSE is a rare genotypic variant of SCD. It has been considered a benign form; however, there are multiple reports of severe complications. Severe complications observed in HBSE disease include vaso-occlusive crisis, acute chest syndrome, stroke, bone marrow embolism, and death.

## Introduction

SCD is a spectrum of hereditary hemoglobinopathies characterized by abnormal hemoglobin S (HbS) polymer. Globally, there are around 3.2 million SCD patients, and 43 million people have sickle cell trait. Out of these, around 176,000 people have fatal complications [1]. The causative mutation in the gene encoding the hemoglobin subunit β (HBB) leads to the formation of various genotypic variants of the disease [2]. This results in a cascade of sickling and unsickling erythrocytes, ultimately leading to hemolysis and/or vaso-occlusion [3]. Common manifestations and complications include but are not limited to hyposthenuria, acute chest syndrome, renal papillary necrosis, painful crisis, pulmonary hypertension, priapism, lower limb ulcerations, osteonecrosis, stroke, and chronic hemolysis [4, 5]. Significant complications such as acute chest syndrome are mainly observed in patients having common genotypes such as HBSS [6].

HBSE, one of the rare genotypes of SCD, has been historically considered to have a benign clinical course in most of cases and is categorized as a mild form of SCD [7, 8]. However, many case reports mention severe vaso-occlusive symptoms in the HBSE genotype, indicating that HBSE might be more severe than thought. Masiello et al. in their concise review described 26 cases of HBSE disease, among whom nine patients had sickling-related complications of varying severity. The review did not report any case with mortality. The authors concluded that HBSE might not be a mild disease, as evident from symptomatic cases [9]. To date, 68 patients with HBSE disease have been reported [7, 9–36]. Many of these had severe complications, and some even died due to the disease manifestations. Management of SCD is expanding, with newly approved disease-modifying drugs such as Voxelotor (1500 mg daily) and Crizanlizumab (5 mg/kg) [37, 38]. Additionally, there are recent trials on gene therapy in SCD with promising effectiveness [39]. Understanding the phenotypic manifestations of less studied SCD genotypes such as HBSE may improve our understanding of the effect of genotype and phenotype interaction on disease severity and suggest targeted therapies. The last review on HBSE was published in 2007; it was a concise review on 26 patients [9]. There have been multiple published cases of HBSE after that, representing variable manifestations of this condition. Many of these reports describe a severe form of SCD, mandating an updated systematic review focused on demographics and manifestations of patients with HBSE disease [10, 15, 24, 28, 30, 33–36]. This review’s main objective is to accumulate all the evidence to date on the demographics, phenotypic manifestations, and complications of the HBSE variant of SCD for its better classification and understanding.

## Materials and methods

### Literature search

A systematic literature search was performed for articles using PubMed, Google Scholar, and Scopus for any date up to January 10, 2021, and all articles in English were analyzed by two authors individually (FA and IK). The following search term was used: “HBSE” OR “Hemoglobin SE.” The extracted articles were screened initially from the title and abstract, and subsequently, a detailed screening was conducted. The quality of the added cases was assessed by two reviewers independently (FA and IK) using the Joanna Briggs Institute case report appraisal checklist for inclusion in systematic reviews [40]. In case of any dispute among the quality assessment, a third reviewer (MAY) independently analyzed the quality of disputed articles to reach a conclusion.

### Study selection

**Fig. 1:**
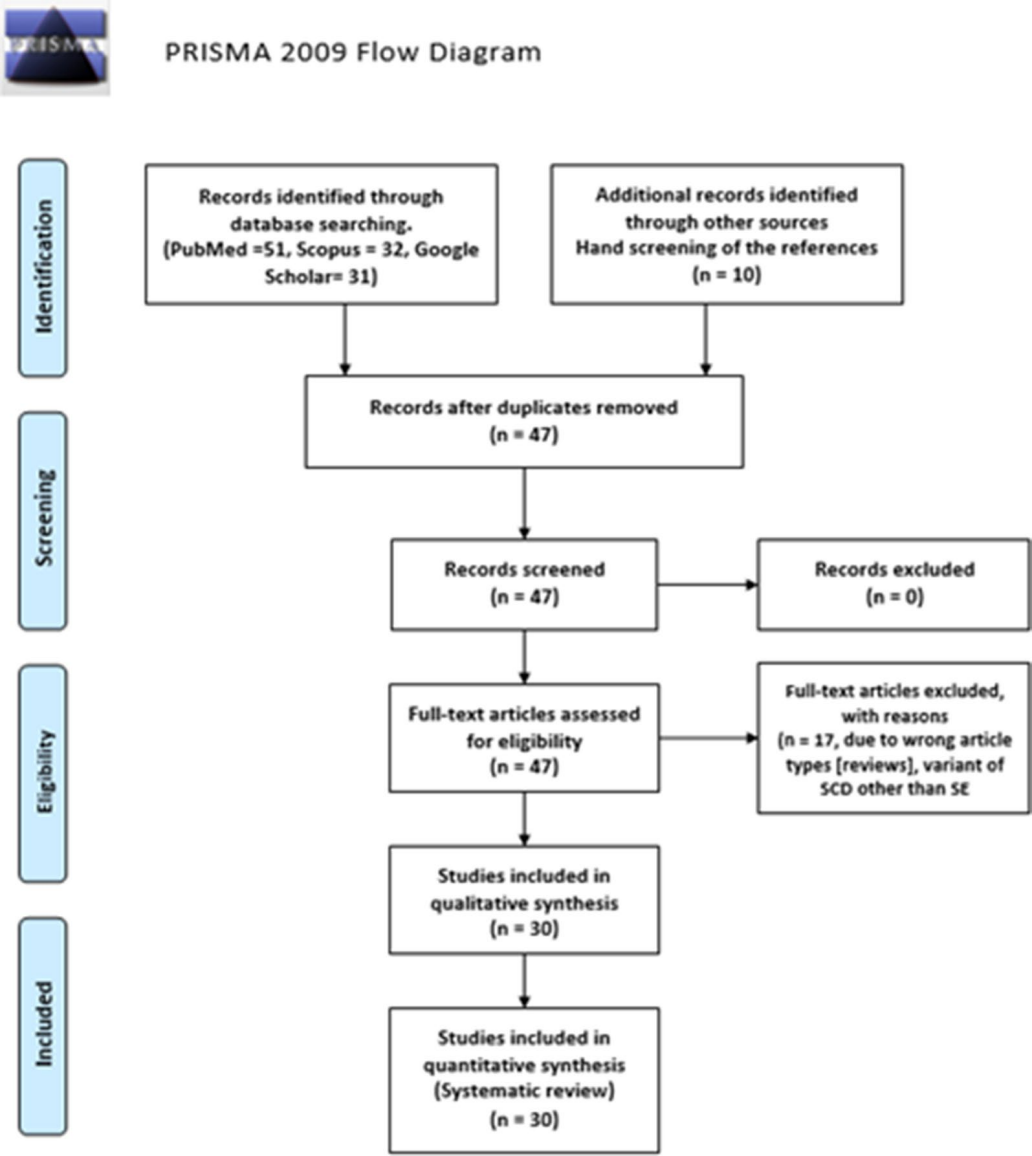
Prisma flowchart with details of the article screening process

Eligible studies (*N* = 30) reported the HBSE genetic variant of SCD worldwide and included case reports, case series, and retrospective studies. Data of 68 HBSE patients were extracted and reported from the finalized 30 articles (Fig. 1). Articles that were not original or reported variants of SCD other than HBSE were excluded from the review.

### Data collection

Epidemiological parameters, clinical pictures, including presenting complaints and complications, laboratory profiles, treatments employed, and outcomes, were noted in all the cases where available. Cases were categorized as mild or severe based on the presence or absence of severe sickling complications, which were infections, functional asplenia, stroke, VTE, bone marrow embolism, splenic sequestration, retinopathy, bone infarct, and acute chest syndrome and death. Severe complications included those which were potentially fatal or had end-organ damage. Data were recorded and analyzed in Microsoft Excel 2016 and SPSS 26.

## Results

We found 68 cases of HBSE in 30 publications (Table 1). Most cases were extracted from case series and retrospective studies (n = 44), while some were found in case reports (n = 24). The highest number of cases were reported from Turkey (n = 22), Oman (n = 13), USA (n = 11), and India (n = 8) in descending order. Males represented 32 (47%) of our cases, whereas females represented 50% ofcases (n = 34). Gender was not mentioned in two cases. The mean age was 20.9 years (SD = 18.2), ranging from 5 months to a maximum of 70 years (Table 2). Most patients had Asian ethnicity (n = 32, 47%), which included South and Southeast Asians, while African lineage (from at least one parent) was found in 8 cases (11.8%). Arabs represented 20.6% (n = 14) of our total cases.

**Table 1:** Reported cases of HBSE to date

Author	Study type	Patients (N) and nationality	*Age (years)*	Hgb (g/dL) and Hb electrophoresis (%)	Manifestations/complications	Treatment	Outcome^a^
			Sex				
Acipayam et al. [10]	RS	20, Turkey	*21.45 ± 15 y*	Hgb: 12.06	3 VOC (1 stroke)	HU = 1	Alive
			8 Males	HBS: 59.5			
			12 Females	HBF: 1.9	17 asymptomatic		
				HBE: 34.5			
Aksoy [11]	CR	1, Turkey	*63 y*	Hgb: 8.4	Splenomegaly	None	Alive
			Female	HBF: 0.8			
Altay et al. [12]	CR	1, Georgia	*20 y*	Hgb: 12.7	NA	None	Alive
			Female	HBS: 64.2			
				HBF: 1.3			
				HBE + HBA2: 34.6			
Andino et al. [13]	CR	1, USA	*24 y*	Hgb: 9	NA	None	Alive
			Female	HBS: 69.8			
				HBE: 30.2			
Arbefeville et al. [14]	CR	1, USA	*12 y*	Hgb: NA	VOC causing ischemia and cardiopulmonary collapse	None	Death
			Male	HBS: 57.4			
				HBE: 34.2			
Baciu et al. [15]	CR	1, USA	*56 y*	Hgb: 13.3	Retinopathy	None	Alive
			Male	HBS: 64.1			
				HBF: 1.2			
				HBE: 34.7			
Bird et al. [16]	CR	1, South Africa	NA	HBE: 28	NA	None	Alive
Eichhorn et al. [17]	CR	1, Turkey	*22 y*	Hgb: 10.8	VOC, pain crisis	Transfusion	Alive
			Female	HBS: 60	Splenomegaly		
				HBE: 40	Parvovirus b19		
Englestad [18]	CR	1, USA	*34 y* Female	Hgb: 10	VOC, pain crisis, chronic pain, hemolytic anemia, pneumonia, acute splenic sequestration, functional asplenia, splenomegaly, retinopathy	Transfusion, splenectomy	Alive
				HBS: 60			
				HBF: 40			
Ganesh et al. [19]	CR	1, Omani	*23 y* Male	HBS: 66		None	Alive
				HBF: 0.5			
				HBE: 33.5			
George et al. [20]	CR	NA	NA	NA	Avascular necrosis	NA	NA
Gupta et al. [21]	CR	1, Pakistani	*28 y* Female	Hgb: 12.9	NA	None	Alive
				HBS: 60			
				HBF: 4			
				HBE: 36			
Gürkan [22]	CR	1, USA	*1 y* Male	Hgb: 10	VOC, pain crisis	None	Alive
				HBS: 58.9			
				HBF: 5.2			
				HBE + HBA2: 37.5			
Hardy et al. [23]	CR	2, Saudi Arabia	*1 y*	Hgb: 11.9	1 VOC	None	Alive
			Male	HBS: 70			
			*6 y*	HBE: 30			
			Female				
Italia et al. [24]	RS	4, India	*20.75 y*	Hgb: 11.05	3 VOC, 1 HA, 1 splenomegaly, 1 chronic pain, pneumonia, ACS	None	Alive
			1 Male	HBS: 58.55			
			3 Females	HBF: 3.6			
				HBE: 26.2			
Ibrahim K et al. [25]	CR	1, Qatar	*17 y*	Hgb: 13.3		HU, transfusion	Alive
			Male	HBS: 67.1			
				HBF: 2.2			
				HBE: 28.7			
Knox-Macaulay et al. [7]	RS	12, Oman	*17.8 y*	Hgb: 12	1 ACS	None	Alive
			8 Males	HBS: 63.5	2 VOC		
			4 Females	HBF: 2.3	1 frontal bossing		
				HBE: 32.7	1 recurrent UTIs		
Masiello et al. [9]	CR	1, USA	*1 y* Male	Hgb: 10	VOC	None	Alive
				HBS: 58.9			
				HBF: 5.2			
				HBE + HBA2: 37.5			
Mishra et al. [26]	CR	2, India	7 y	Hgb: 9.3	Splenomegaly	None	Alive
			Male	HBS: 58.05	HA		
			*20 y*	HBF: 2.8			
			Female	HBE + HBA2 = 35.4			
Mukhopadhyay et al. [27]	CR	1, India	*26 y* Male	Hgb: NA	NA	None	Alive
				HBS: 58.1			
				HBF:4.3			
				HBE: 33.2			
Pajak et al. [28]	CR	1, USA	*55 y* Female	Hgb: 11.7	HA, VOC, splenomegaly, functional asplenia	Splenectomy	Alive
				HBS: 68.6			
				HBF: 1.4			
				HBE: 26.3			
Ramahi et al. [29]	CR	1, USA	*28 y* Female	Hgb: NA	Postpartum endometritis	Transfusion	Alive
				HBS: 65.3			
				HBF: 3.5			
				HBE: 31.2			
Rayburg et al. [30]	CR	1, USA	7 y	Hgb: 11.4	VOC, HA, bone marrow infection with parvovirus b19, bone marrow embolism, PE, Splenomegaly asymptomatic	None	Death
			Female	HBS: 55			
				HBF: 1.3			
				HBE: 31			
Rey et al. [31]	RS	4, Haiti	*4.1 y*	Hgb: 11.4		None	Alive
			2 Males	HBS: 61.5			
			2 Females	HBE + HBA2: 30			
Schroeder et al. [32]	CR	1, USA	*22 y*	Hgb: 14.6	Hematuria	None	Alive
			Male	HBS: 60	Splenomegaly		
				HBE + HBA2: 32			
Smith et al. [33]	CR	1, USA	*52 y*	Hgb: 13.4	VOC, HA, Gallstones	Transfusion	Death
			Female	HBS: 25			
				HBE: 8			
Tamminga et al. [34]	CR	1, Netherlands	7 y	Hgb: 6.8	Rhabdomyolysis	None	Alive
			Male	HBS: 65			
				HBF: 1.4			
				HBE: 34			
Tay et al. [35]	CR	1, Bangladesh	*66 y* Female	Hgb: 9.3	HA, VOC, ACS, AVN	Transfusion, HU	Alive
				HBS: 61.2			
				HBF: 7.2			
Thornburg et al. [36]	CR	1, USA	*2* Male	Hgb: 10.2	NA	NA	Alive
				HBS: 68			
				HBE: 32			
Vishwanathan et al. [47]	CR	1, India	*18 y* Male	Hgb: 9	VOC	NA	Alive
				HBS: 68	HA		
				HBF: 2.1	Splenomegaly		
				HBE: 29.9			

*Hgb* hemoglobin, *HU* hydroxyurea, *SCT* stem cell transplant, *VOC* vaso-occlusive crisis, *HA* hemolytic anemia, *ACS* acute chest syndrome, *AVN* AVASCULAR necrosis, *UTI* urinary tract infections, *PE* pulmonary embolism, *RS* retrospective study, *CR* case report, *NA* not available

^a^Outcome at the time of the case report/series

**Table 2:** Demographics of SCD patients with HBSE genotype compared to classic SCD [46]

Characteristics	HBSE reported cases, N = 68
Age^a^ (years)	Mean: 20.9 ± 18.26 years
HbS (percentage)	Mean: 61.1 ± 7.25
HbE (percentage)	Mean: 32.3 ± 5.06
HbF (percentage)	Mean: 2.2 ± 2.16
Hemoglobin (g/dl)	Mean: 11.64 g/dl ± 1.73
	Mean (males): 11.69
	Mean (females): 11.61
MCV (fl)	Mean: 70.5 ± 6.01
Presentation (diagnosis)	Asymptomatic at presentation: 31 (45%)
	Presentation at birth: 2 (2.8%)
	Discovered in Clinic: 1 (1.4%)
	Diagnosed during hospitalization: 8 (11.8%)
	Discovered Postmortem: 2 (2.8%)
Asymptomatic cases (no complications found)	*N*: 35 (51.5%)
Treatments given	Transfusion (exchange or simple): 7 (10.2%) Hydroxyurea: 3 (4.4%)
Death due to complications	*N*: 3 (4.2%)

^a^Age at presentation

**Fig. 2:**
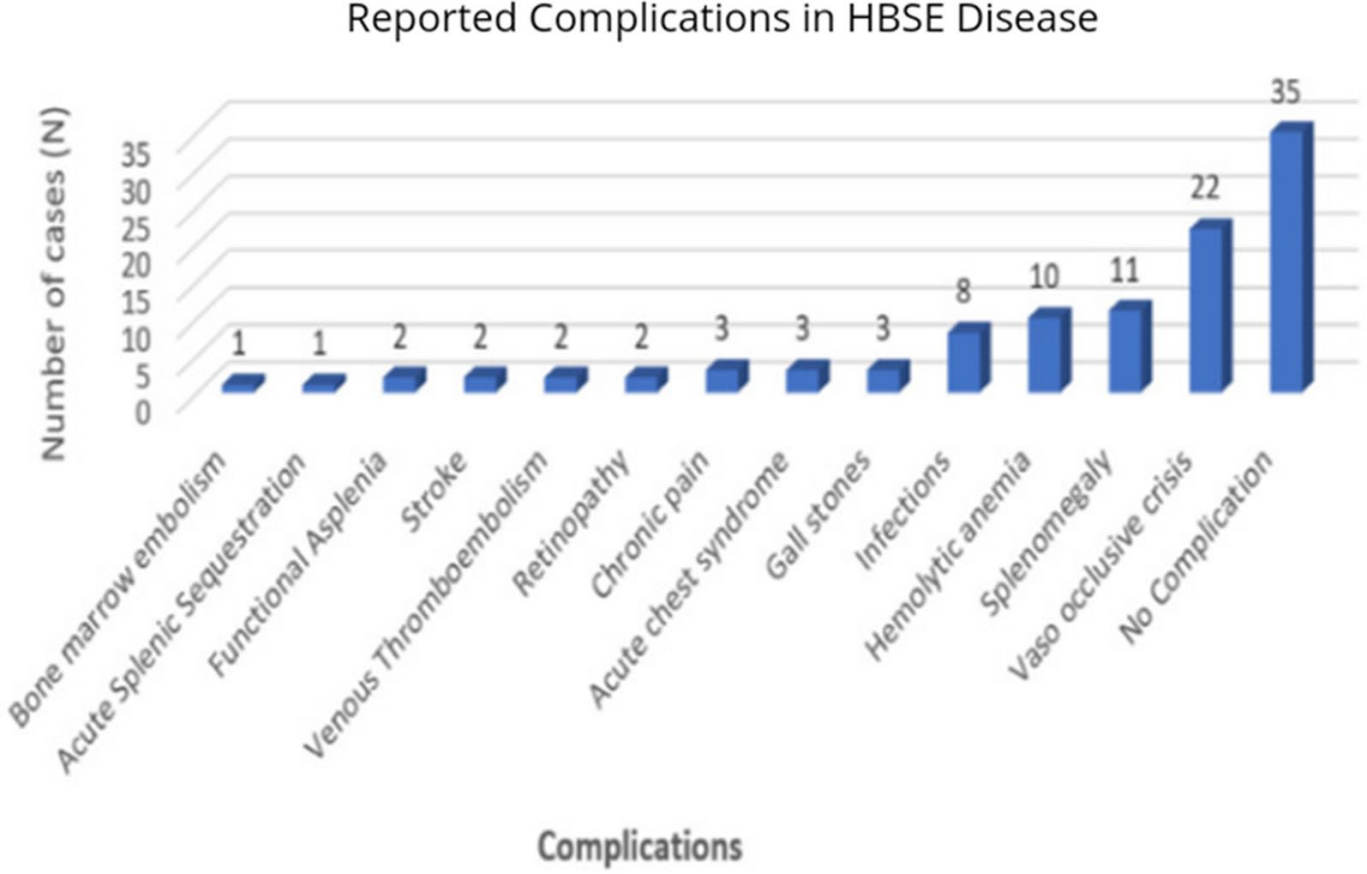
Reported complications in HBSE disease

Acute vaso-occlusive pain crisis was the most common complication reported by 22 cases (32.3%), with splenomegaly (n = 11, 16.1%), hemolytic anemia (n = 10, 14.7%), infections (n = 8. 11.8%), bone infarction (n = 4, 5.9%) representing other common complications (Fig. 2). Respiratory manifestations (thoracic pain, cough, bronchitis, dyspnea, or upper respiratory infection) were reported in five cases (7.3%). Gallstones were seen in three cases (4.4%). Venous thromboembolism (VTE) (2.9%) and stroke (2.9%) were both described in two separate cases each. Hematuria was also present in two patients (2.9%).

We did not find any report describing cardiopulmonary complications (pulmonary hypertension, myocardial infarction, cardiomyopathy), which commonly affect SCD patients. Other serious renal complications were not reported in any of the 68 cases in the literature.

Most of the reported patients had mild to moderate disease. However, mortality due to complications was documented in three cases. Out of those three patients, the first patient, a 52-year-old female, had an undiagnosed potentially uncomplicated disease and revealed only postmortem. The disease rapidly deteriorated, leading to venous thromboembolism and cardiac arrest within 26 h of admission. The cause of death was thought to be ischemia due to dehydration that led to a sickling crisis [33]. The second death occurred in a 12-year-old male within 24 h of symptom onset due to sudden cardiopulmonary collapse due to vaso-occlusion leading to ischemia [14]. The third fatality occurred in a 7-year-old female with a history of multiple hospitalizations with pain crises, hemolytic anemia, and Parvovirus B19 infection. Although she had a prolonged and complicated course of her disease, her deterioration before death was acute, secondary to a massive marrow embolism in the pulmonary circulation [30].

Mean hemoglobin (Hgb) was 11.64 mg/dl, slightly higher in males (11.69 mg/dl) than females (11.61 mg/dl). Mean percentages of hemoglobin S (HbS), hemoglobin E (HbE), and fetal hemoglobin (HbF) are given in Table 2. Average MCV was low (70.9 fL), with minimum and maximum reported being 57.9 fl and 82 fl, respectively.

## Discussion

This study represents an updated comprehensive systematic review on demographics and manifestations of HBSE genotypic variant of SCD. The genetic basis of HBS lies in glutamine to valine, at position six in the beta-globin gene, whereas that of HBE lies in the substitution of lysine for glutamine at amino acid number 26, in the beta-globin gene [41, 42]. HBSE variant occurs as a result of a combination of HBS and HBE genotypes [28]. Hemoglobin E trait and disease are not uncommon and are second only to the HBS spectrum in global prevalence [43]. However, the combination of two different abnormal mutations makes HBSE a rare occurrence.

The exact incidence of the HBSE genotype remains unknown. A retrospective review of 12 patients in Oman mentioned a point prevalence of HBSE heterozygotes at 0.05%. The authors also concluded that given a very small fraction of HBSE patients developing symptoms, it is a very mild condition, and severe sickling complications are rarely seen [7]. However, in our review, we found a wide spectrum of SCD complications ranging from asymptomatic patients (51.5%) to death (4.4%). Complications included VOC pain crisis (32.3%), splenomegaly (16.1%), hemolysis (14.7%), infections (11.8%), bone infarction (5.9%), cholelithiasis (4.4%), thromboembolism (2.9%), stroke (2.9%), and hematuria (2.9%).

In a review on HBSE disease published in 2007, David Masiello et al. found that among the 26 patients with HBSE disease, nine patients aged 18 or younger were generally well. However, more than half of the patients older than 18 presented with sickling-related complications. They attributed this to the years of accumulating sickling vasculopathy that makes complication rate increase with aging [9]. We analyzed the complications based on age by dividing them into below 18 and above 18 years in cases where ages were specified for the complications (22 children and 24 adults). Overall, the complication rate in adults was higher than the pediatric age group, which is in line with the previous review of 16 patients. The only significant difference in complications based on age was found in the occurrence of hemolytic anemia, which was present in 4.5% (1/22) of cases < 18 years old compared to 37.5% (9/24) of cases > 18 years of age (OR 12.6, P-value = 0.011). The low incidence of hemolytic anemia in children with HBSE can be related to the presence of fetal hemoglobin, which has a protective effect against many of the complications secondary to sickling, including death [8]. Acute vaso-occlusive pain crisis was seen in 31.8% (7/22) children (age < 18 years) compared to 37.5% (9/24) adults. On the other hand, chronic pain was slightly more common in adults; 4.5% (1/22) of pediatric cases compared to 8.3% (2/24) of adult cases. Pain crisis was not considered a severe complication of SCD, but it is a clinically significant symptom for the patient due to the type of sensation involved. Additionally, in patients > 20 years of age, pain frequency has been described to be associated with early mortality [8]. Those patients who presented with gallstones (n = 3), retinopathy (n = 2), functional asplenia (n = 2), and splenic sequestration (n = 1) were adults. The infection rate was comparable in both age groups; 13.6% (3/22) in children and 16.7% (4/24) in adults. Splenomegaly was present in 18.2% (4/22) of pediatric cases compared to 29.2% (7/24) of adults. Bone infarction and acute chest syndrome were present in 4.5% (1/22) of children compared to 8.3% (2/24) of adults. Bone marrow embolism was reported in one pediatric case only. VTE was present in one pediatric and one adult patient. Mortality, however, was seen more in children (two cases) compared to adults (one case).

We found a slight female predominance in the HBSE population (50%) compared to males (47%). It is to be noted that gender was not reported in two patients, which could have increased or decreased the asymmetry between both genders. Our finding, although less disproportionate, is in keeping with a previous review on 27 patients with HBSE disease, where the authors described 73.6% female cases. In their review, Mishra P et al. reported that 40.7% of the total patients were symptomatic [26]. In our review, 35 (51.5%) of the 68 patients had no complications reported, while 48.5% suffered from mild, moderate, or severe SCD related complications. Although high levels of HBF are considered protective against SCD complications, no such correlation was found by Mishra et al. in their review [26, 44]. To analyze the association of HBF with the severity of complications, we divided the complications into mild-moderate and severe. The mean HBF level in those with mild to moderate complications was higher than those with severe complications, including death (2.4% versus 1.9%). However, no statistical significance was found. A better understanding of this patient-to-patient clinical variability in more extensive studies would dramatically improve care and guide the development of novel therapies. Studies of the natural history of these β-hemoglobinopathies have identified fetal hemoglobin levels and concomitant α-thalassemia as important modifiers of disease severity. Fortunately, improved knowledge of the human genome and the development of new genomic tools, such as genome-wide genotyping arrays and next-generation DNA sequencers, offer new opportunities to use genetics to understand better the causes of the many complications observed in β-hemoglobinopathy patients [45]. Correlation of the clinical and hematological features of HbSE cases with their α-globin gene status and β-cluster haplotypes merits additional considerations in future studies to better understand phenotypes and the disease modifiers and probably novel agents.

Turkey is a country with a higher prevalence of hemoglobinopathies such as HBE and HBS with significant population admixture and racial intermarriages. One retrospective study on 20 patients with HBSE disease reported a mean Hgb of 12.06 g/dl, which is slightly higher than what we found in our review (11.64 g/dl). The study reported a mean MCV of 69.9 fl, which on the other hand, is slightly lower than the one in our review, 70.9 fl. 15% of the patients in that study had sickling-related complications compared to 68% in our review [10]. The increased published reports of complications related to HBSE reveals the potential morbidity and mortality related to the genotype. Similar to HBSS, HBSE genotype results in a range of phenotypes and may be fatal. There may be subclinical or long-term subtle complications of HBSE that have not yet been well described. The increasing evidence emphasizes early detection and close follow-up of HBSE patients for better patient management. Additionally, it is imperative to reclassify HBSE disease as a moderate form of SCD rather than mild.

As it is a variant of sickle cell disease, the clinical features and biochemical profile of HBSE patients are comparable to the classic HBSS SCD. However, there are some notable differences. In our review, we found the mean age at presentation with symptoms 20.9 years, whereas the median age of presentation in classic SCD is reported around 36 years in a previous study [46]. Another difference that we appreciated is in mean HBF percentage, which is 2.2% in HBSE patients, and 5% in HBSS patients. Lastly, because management of HBSE disease is not well-established, the use of HU in HBSE patients is considerably low (4.4%) compared to HBSS patients (39%) [46]. A direct comparison of both types of SCD in a similar patient population in extensive studies can validate our findings.

Over the years, many treatment modalities have been developed for SCD, among which hydroxyurea and simple or exchange blood transfusion have an established role in treating or preventing complications. Curative management includes historical treatment such as stem cell transplantation and new and upcoming gene therapy [3]. Some newer management drugs that have recently been approved for the treatment of SCD include Voxelotor and Crizanlizumab [37, 38]. In our review, blood transfusion and hydroxyurea have been used in few patients and improved their conditions [10, 17, 25, 29, 33, 35].

Our review has some limitations inherent when conducting systematic reviews of rare conditions. Firstly, around 30 percent of the data was extracted from case reports. Secondly, missing data from case reports and retrospective studies limited an extensive data analysis. Thirdly, we acknowledge that publication bias may have contributed to the increased number of complications reported in recent years. Many patients with asymptomatic course who do not require medical attention would go unnoticed. The lack of systematic prospective data precludes a proper understanding of the actual rates, incidence, or timeline of the development of major or minor complications. Lastly, as it is challenging to distinguish HBSE from other variants of SCD, such as HBSC on readily available tests, a bias in reporting HBSE as HBSC (or other variants) could be present in the literature, decreasing the total number of reported HBSE cases.

This review opens the door to consider various SCD therapies in managing HBSE patients with severe or recurrent complications. More extensive studies on the HBSE patient population must formulate management guidelines to treat its moderate to severe complications. Hydroxyurea, the mainstay of treatment of sickle cell disease, has a well-established role in HBSS and Hb SB^+^ [9]. However, due to the rare occurrence of HBSE, there are no trials available for the use of HU in this variant of SCD. In our review, HU was used in 4.4% of patients, with good outcomes. Establishing a definite role of HU in symptomatic patients with HBSE might take years as the incidence of HBSE itself, and then the prevalence of symptoms in this patient population is still too low to analyze the use of HU in HBSE prospectively. Meanwhile, we suggest using HU in HBSE patients with moderate to severe VOC and acute chest syndrome symptoms on a case-to-case basis until more extensive evidence becomes available.

## Conclusion

HBSE disease has been considered as a very benign variant of SCD with minimal to no sickling symptoms. However, a growing literature on HBSE and its complications suggests that HBSE considerable number of patients have a moderate form of SCD with a wide range of significant complications, including mortality. More extensive studies with additional data can help categorize and understand HBSE disease and its manifestations better.

## Data Availability

Data sharing not applicable.
